# [Expression of Concern] Luteolin protects mice from severe acute pancreatitis by exerting HO-1-mediated anti-inflammatory and antioxidant effects

**DOI:** 10.3892/ijmm.2026.5939

**Published:** 2026-07-27

**Authors:** Jie Xiong, Kezhou Wang, Chunxiao Yuan, Rong Xing, Jianbo Ni, Guoyong Hu, Fengling Chen, Xingpeng Wang

Int J Mol Med 39: 113-125, 2017; DOI: 10.3892/ijmm.2016.2809

Following the publication of the above paper, it was drawn to the Editor's attention by a concerned reader that, regarding the histological images shown in Fig. 2A on p. 117, the 'NC/3 h' and 'NC/6 h' data panels contained an overlapping section of data, suggesting that these data panels were derived from the same original source where different experimental conditions were reported.

The authors have been contacted by the Editorial Office to offer an explanation for this apparent anomaly in the presentation of the data in their paper, and we are awaiting their response. Due to the fact that we have been made aware of potential issues surrounding the scientific integrity of this paper, we are issuing an Expression of Concern to notify readers of this potential problem while the Editorial Office continues to investigate this matter further.

